# The Pathogenesis of Glenohumeral Deformity and Contracture Formation in Obstetric Brachial Plexus Palsy—A Review

**DOI:** 10.1055/s-0039-1692420

**Published:** 2019-07-12

**Authors:** Pontus N. Olofsson, Alice Chu, Aleksandra M. McGrath

**Affiliations:** 1 Department of Hand and Plastic Surgery, Norrland's University Hospital, Umeå, Sweden; 2 Department of Surgical and Perioperative Sciences, Umeå University, Umeå, Sweden; 3 Department of Orthopedic Surgery, NYU Langone Orthopedic Hospital, New York, New York, United States; 4 Department of Clinical Science, Umeå University, Umeå, Sweden

**Keywords:** obstetric brachial plexus palsy, brachial plexus, shoulder contracture, glenohumeral joint dysplasia, internal rotation contracture

## Abstract

Contractures of the shoulder joint and glenohumeral joint dysplasia are well known complications to obstetrical brachial plexus palsy. Despite extensive description of these sequelae, the exact pathogenesis remains unknown. The prevailing theory to explain the contractures and glenohumeral joint dysplasia states that upper trunk injury leads to nonuniform muscle recovery and thus imbalance between internal and external rotators of the shoulder. More recently, another explanation has been proposed, hypothesizing that denervation leads to reduced growth of developing muscles and that reinnervation might suppress contracture formation. An understanding of the pathogenesis is desirable for development of effective prophylactic treatment. This article aims to describe the current state of knowledge regarding these important complications.

## Introduction

Obstetric brachial plexus palsy (OBPP) due to traction injury to the nerves of the upper extremity occurs in 1.5 to 5.1/1,000 births. 1 2 3 4 OBPP is often self-limiting with reported spontaneous recovery rate of 66 to 82%. 2 3 4 7 8 Different rates could be attributed to varying definitions of recovery, however, recent review suggests that the rates may be overestimated. 4 5 6 7 Pondaag et al concluded that studies on recovery rates conducted before 2001 were methodologically insufficient. 7

Despite neurologic recovery, longstanding and disabling sequelae caused by glenohumeral joint dysplasia (GHD) and contractures of the shoulder are common. GHD is a set of skeletal alterations in the glenoid cavity and in the humeral head secondary to OBPP, which reversibility has not been clearly defined. 9 10

These secondary changes of the glenohumeral joint could limit the beneficial results of primary nerve reconstruction and are, together with contractures, a common indication for secondary reconstructive surgery. Thirty-three percent of all OBPP patients, and as many as 58 to 74% of patients examined with shoulder magnetic resonance imaging (MRI) prior to nerve surgery because of higher severity of OBPP, had GHD of some degree. 11 12 13

Contractures are defined as a decreased range of movements of the joint due to changes in nonskeletal tissues. Hoeksma et al reported that 56% of all OBPP patients develop shoulder contractures and that, once established, shoulder contractures do not resolve spontaneously. 11

Though not a subject of this review, elbow flexion contracture is also common. A retrospective study of 319 patients with various degree of OBPP found a 48% prevalence of elbow flexion contracture. 14

A clear understanding of the pathogenesis behind these complications is lacking. Until recently, the prevailing theory for causation has been muscle imbalance between internal and external rotators, as well as abductors and adductors of the shoulder. 15 16 17 18 19 Although theoretically compelling, it fails to explain some of the findings in OBPP patients. Several newer studies have presented other theories, which challenge the muscle imbalance hypothesis. The ultimate aim of these studies is providing better understanding of these complications to develop more effective treatment strategies and preventive measures. 12 This article will review existing literature regarding the pathogenesis of GHD and its relationship to contractures, severity of OBPP, and muscle atrophy.

## Classification of Glenohumeral Joint Deformity—Defining the Concepts

**Fig. 1:**
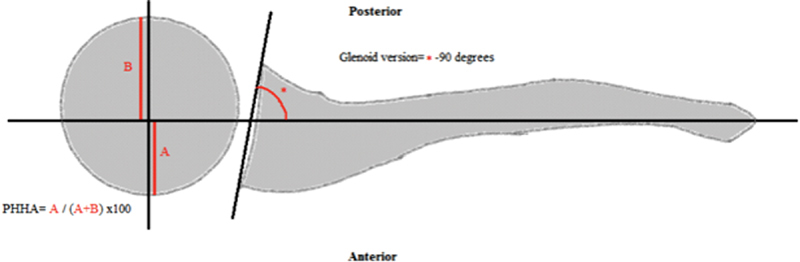
Diagram depicting the method of measuring percentage of humeral head posterior anterior to the middle of the glenoid fossa (PHHA), and the glenoid version according to Friedman et al. 18 For PHHA, the length of the humeral head anterior to the middle of the spine of scapula is divided by the diameter of the humeral head. For glenoid version, the angle between the spine of scapula and the glenoid surface is measured and subtracted by 90 degrees.

Much attention has been given to the deformation of bony structures around the glenohumeral joint. After MRI and computed tomography studies on OBPP patients, Waters et al presented a grading system from I to VII to classify frequently seen patterns of deformity ( Table 1 ). 15 The percentage of humeral head anterior to the middle of the glenoid fossa (PHHA) and the glenoid version, measured according to Friedman et al 20 ( Fig. 1 ), are central in grading. For PHHA, a line is drawn along the spine of scapula, bisecting the humeral head. The distance from this line to the anterior aspect of the humeral head is divided by the total diameter of the humeral head. This ratio represents the proportion of humeral head anterior to the spine of scapula ( Fig. 1 ). Presence of a false glenoid and the shape of the glenoid and humerus are also considered. Pearl and Edgerton classified four different shapes of the glenoid in OBPP patients: concentric, flat, biconcave, or pseudoglenoid. 21 Birch presented a similar classification with the three grades; concave-flat, convex, and biconcave. 22 The above-mentioned deformities can arise as early as within the first 3 months of life, 12 and severity progresses with age. 23 Other findings in OBPP patients include smaller humeral head, loss of humeral head symmetry, and flattening of the anterior aspect of the humeral head. 19 24

**Table 1:** Classification of GHD according to Waters

	Waters' classification 13
Grade	Findings
I	Normal glenoid (< 5 degrees difference in retroversion compared with normal, contralateral, side)
II	Minimum deformity (> 5 degrees difference in retroversion and no posterior subluxation of humeral head)
III	Moderate (posterior subluxation < 35%)
IV	Severe (pseudoglenoid formation)
V	Severe flattening of humeral head and glenoid, with progressive or complete posterior dislocation of humeral head
VI	Glenohumeral joint dislocation in infancy
VII	Growth arrest of proximal humerus

The biomechanics of the pathologic force vectors acting upon the glenohumeral contact area, which are likely to be involved in development of the shoulder deformity (the backward movement of the humeral head occurring with shoulder internal contracture), have been studied, including pilot studies employing multifactorial motion analysis to combine range of motion (ROM), muscle activity, forces, and torque acting on the glenohumeral joint in children with OBPP. 10 25

## Clinical Studies

### Correlation of Glenohumeral Joint Deformity to Contractures

It has been proposed that a shoulder internal rotation contracture leads to GHD by keeping the humeral head in an internally rotated position, causing excessive stress on both the humeral head and glenoid as they grow. 16 19 26 Several studies have found correlation between contractures and bony deformities. 11 18 19 21 24 Kozin found that, in his 33 patients with residual brachial plexopathy (average 4.9 years of age, global lesions excluded), all children with internal rotation contracture had some degree of GHD, and the degree of restriction of passive external rotation correlated with more glenoid retroversion, PHHA, and deformed glenoid shape. A clear definition of internal rotation contracture is not provided in this study. However, Kozin stipulated that a clinical exam with shoulder external rotation < 0 degree should be regarded as an indicator for GHD. 19

Pearl and Edgerton also noted a correlation between the degree of internal rotation contracture and severity of GHD in their study on 25 patients (age 1.5–13.5 years) undergoing secondary reconstruction for internal rotation contracture. GHD was defined as abnormal glenoid shape as seen on intraoperative arthrographs. In contrast to the prior study, they found that not all shoulders with an internal rotation contracture developed GHD, and they therefore concluded that other factors besides contracture play a part in deformity formation. 21 The methods for imaging could contribute to this difference. While Kozin used MRI to assess for the presence of GHD, Pearl and Edgerton's study was based on intraoperative arthrography performed with an abducted shoulder. This centers the caput in the glenoid, while adduction and internal rotation while performing MRI might accentuate GHD when defined by PHHA. Hoeksma et al also found a strong correlation for shoulder contracture > 10 degrees and GHD (smaller humeral head, abnormal clavicle, or abnormal glenoid fossa) based on radiographs in a group of 4-year-olds with various degree of OBPP. 11

In contrast to the mentioned findings, 19 21 Waters et al could not find a correlation between passive shoulder external rotation and GHD, defined according to Waters' classification on MRI. The mean age was 2.5 years (range: 7 months–6.2 years). 27 Lack of correlation is a finding shared by van Gelein Vitringa et al in a prospective MRI study on 24 children (mean age: 3.3 years, range: 14.7 months–7.3 years). No correlation between passive external rotation and glenoid version, posterior subluxation, or Birch's classification could be found. 28 Interestingly, Iorio et al found GHD, as classified according to Waters et al, 15 in patients without decreased passive external rotation. 12 This study consisted of 19 patients with preoperative MRI scans before undergoing surgical exploration for nerve reconstruction. The study population was notably younger than the other studies described in this section, median age 16 weeks (interquartile range: 14–46 weeks), and it is possible that contractures would develop with age in patients with GHD.

Age and contractures could theoretically work together to form GHD due to a longer period with the shoulder internally rotated. However, the finding of GHD preceding contractures challenges this theory. While some of the studies indicate that contractures and GHD are associated and possibly the first might be a cause of the latter; other studies fail to find this association, and in one of the studies the presence of GHD was found without limited passive ROM. We believe that there may be other, separate, mechanisms causing these two complications.

### Correlation between GHD and Extent of OBPP

To grade the extent of nerve root involvement in OBPP, the Narakas classification is commonly used. Type 1 is an injury to the C5-C6 roots (Erb's palsy). Type 2 involves C5-C7 (extended Erb's), type 3 involves the whole brachial plexus (total palsy), and type 4 is a total palsy accompanied by Horner's syndrome. 29 Theoretically, Erb's paralysis or extended Erb's would lead to paralysis of the external rotators but preservation of some internal rotation force, yielding a greater muscle imbalance than total paralysis. If muscle imbalance is the main factor causing contractures and GHD, we would hypothesize an inverse correlation between GHD severity and extent of OBPP.

Iorio et al found Erb's palsy to correlate with more severe GHD compared with total palsies in their MRI study on 16 (14–46) weeks old patients. They also found normal shoulders in 50% of patients with Narakas grade 4 palsies, while the other 50% had substantial GHD. The patients with substantial GHD had early recovery of internal rotation potentially leading to rotational imbalance, which might explain the findings. 12 Iorio et al's findings are supported by Hogendoorn et al who found C5-C6 (±C7) injuries to have more glenoid retroversion and posterior humeral head subluxation compared with total palsies in 102 patients (5 ± 3.4 years of age) with residual impairment. However, no difference between extent of OBPP was found when assessing glenoid and humeral head shape, or rotator cuff atrophy on MRI. 26

Other authors have reported no correlation between extent of OBPP and GHD. van der Sluijs et al could not establish a causality in an MRI study on 26 infants 5.6 (2.7–14.5) months old considered for primary nerve repair due to lack of progression of neurological recovery. In this study, GHD was assessed by measuring glenoid version, glenoid shape according to Birch, and posterior subluxation. 13 Hoeksma et al also found that Narakas classification at birth cannot predict future grade of GHD, defined as nonspherical humeral head or abnormal glenoid on radiographs. 11 Waters et al found that patients with higher Narakas grades have more severe GHD than partial lesions. 27

In summary, studies investigating the correlation between GHD and Narakas grade reach different results. Likely, the effect of spontaneous reinnervation and primary nerve surgery affects the results in unpredictable ways. We cannot conclude that the pattern of denervation has a predictable outcome regarding GHD.

## Muscle Atrophy and Its Relation to Glenohumeral Joint Dysplasia and Contractures

Atrophy of the rotator cuff muscles, most notably atrophy of the subscapularis, has been linked to GHD. 18 26 28 Einarsson et al found the subscapularis to be structurally different in nine patients with shoulder internal rotation contracture. Single muscle fibers had shorter slack sarcomere length and increased stiffness, hypothesized to be due to lack of passive stretch on the subscapularis from the injured external rotators. However, the control muscles in this study were other upper extremity muscles and not subscapularis muscle, which could affect results. 30 A later study showed normal histological appearance in 12 of 13 subscapularis specimens from OBPP patients undergoing secondary surgery for shoulder internal contracture, although sarcomere lengths and stiffness were not measured in that study. Instead, the type of myosin heavy chain, ratio of muscle cells to extracellular matrix, fibrosis, and muscle fiber diameter were assessed. 31

Pöyhiä et al conducted a MRI study on 42 children (mean age: 7.7 years) with shoulder internal rotation contracture, lack of active shoulder external rotation, or possible posterior subluxation of the humeral head. Atrophy was measured from the oblique sagittal plane using the greatest thickness of the supraspinatus, infraspinatus, and subscapularis muscles compared with the uninjured side. In this study, infraspinatus and subscapularis were equally atrophic. Muscle atrophy was correlated to passive external rotation, which in turn correlated to glenoid version and PHHA. An increased difference in muscle thickness between injured and uninjured side for subscapularis and infraspinatus muscle thickness correlated to glenoid version and PHHA. 18

van Gelein Vitringa et al discovered atrophy of both infraspinatus and subscapularis, with subscapularis the most affected. 28 This study included 24 patients with internal rotation contracture considered for secondary reconstructive surgery. Population age was younger than Pöyhiä et al's (mean age: 3.3 years). While Pöyhiä et al's study included 6 patients (15%) with previous subscapular release procedure, van Gelein Vitringa et al excluded patients with previous secondary reconstructive surgery, but both studies included patients who had undergone primary nerve repair. In van Gelein Vitringa et al's study, unaffected shoulders' as well as affected infraspinatus' muscle volume increased with age, with the exception of subscapularis muscle volume on the affected side. Atrophy of the infraspinatus and subscapularis was associated with posterior subluxation but not with glenoid version or Mallet score. Notably, there was no relation between muscle atrophy and passive external rotation nor passive external rotation and GHD. 28

This was surprising since, in theory, the craniocaudal distribution of nerve root injury would cause greater denervation of the infraspinatus and consequently greater atrophy. The hypothesis proposed for this finding is similar to Einarsson et al's, and assumes that insufficient lengthening from external rotators may cause atrophy of the subscapularis by positioning the subscapularis in a shortened and immobilized state. 30 This theory was confirmed by animal studies showing that changes in length adaptation shown after denervation might result from a myogenic mechanism rather than neurogenic one. The morphological changes confirmed result in a loss of sarcomere numbers and muscle belly shortening because of immobilization in shortened position rather than alterations in sarcomere length. 32 A study on a rodent model showed that denervation of external rotators with intact subscapularis innervation lead to atrophy of the subscapularis. 32

While the reasons for the inconsistent findings in these two MRI studies are unclear, differences of the cohorts and methods of assessing the atrophy are possible factors. Pöyhiä et al measured the greatest thickness of the muscles. van Gelein Vitringa et al did so to enable comparison, but they also measured the area of the muscles in the standardized transverse plane sections. We believe the latter method to measure muscle atrophy is more reliable since muscle thickness could vary greatly depending on arm positioning. 18 28

Hogendoorn et al had a study population of 102 patients with external rotation < 0 degree or Mallet score of < 3 for hand-to-mouth and/or hand-to-head movements. The patients were 5 ± 3.4 years old, and none had undergone secondary reconstruction. 26 The authors measured the muscle diameter in a single transverse section and evaluated the presence of degenerative fatty infiltration on a three-graded scale. If the diameter on the affected side was smaller, the muscle was graded as atrophic. This study found that atrophy of all rotator cuff muscles was correlated to PHHA, as well as the glenoid shape. 26 The subscapularis was the most degenerated muscle and degeneration of the subscapularis muscle was the only muscular change related to glenoid version. In contrast, Pöyhiä et al found glenoid version to be correlated to atrophy of all rotator cuff muscles. 18

In an MRI study on 74 children (2.5 ± 1.2 years old) with no secondary reconstructive procedures and residual OBPP and GHD of various degrees, Waters et al found the difference between pectoralis major and subscapularis cross-sectional areas (measured in a single standardized axial image) compared with external rotators to be greater in OBPP shoulders than contralateral control limbs. The ratio between internal and external rotators was linked to GHD (Waters' classification) and a higher ratio was related to worse deformity. Interestingly, in this study a higher Narakas grade was linked to worse GHD and passive shoulder external rotation in adducted position did not correlate to GHD. 27

In summary, two studies have found the subscapularis to be the most atrophic shoulder muscle. 26 28 If lack of external rotation force were the cause of subscapularis atrophy, muscle imbalance could explain that finding as well. However, these studies show conflicting results regarding correlation between Narakas grade and GHD severity, as well as the correlation between passive external rotation and atrophy, and external rotation and GHD. Narakas grade was not reliably related to atrophy. 26 28 Atrophy of rotator cuff muscles on the affected side is common and expected; however, we believe that there are other muscular changes responsible for GHD. The inconsistencies presented, in our opinion, might indicate that there are other factors than muscle imbalance solely, responsible for causing contractures and GHD.

## Anatomic Pathogenesis of Glenohumeral Joint Dysplasia

Since many studies have found a correlation between contractures and GHD and the prevailing hypothesis for contractures has been the strength imbalance theory, many studies draw the conclusion that GHD as well is caused by strength imbalance. 12 15 16 19 21 33 A much less likely etiology would be direct joint trauma during birth, as seen in adult reports of shoulder fracture dislocation with accompanying brachial plexus palsy. 34 A stipulated mechanism is that, in OBPP patients, the abnormal posture of the upper extremity results in excessive force on the posterior part of the glenoid leading to erosion and/or inhibition of growth in that part of the glenoid, subsequently leading to pseudoglenoid formation and posterior dislocation. 21 The Hueter–Volkmann law, which stipulates that decreased stress on a growth plate increases bone formation, and excessive stress decreases bone formation, has been proposed as a hypothesis explaining GHD in OBPP. 19 It likely works in concert with principles of bone development described by Pauwels, who noted that the functional adaptation of bone growth is related to mechanical stress. 35 36

This theory was only partly confirmed by van Gelein Vitringa et al. 37 They hypothesized that posterior dislocation would lead to excessive forces on the dorsal side of the scapula and decreased forces on the ventral side. The ratio of ventral to dorsal side length of the scapula was significantly correlated to the grade of posterior subluxation. Consistent with the Hueter–Volkmann law, this study found a decreased length of the dorsal side of the scapula but failed to find the reciprocal length increase of the ventral side of the scapula. 37 In a similar way, Pearl et al found the anterior part of the humerus flattened in patients with internal rotation contracture. The contracture positions that part of the humerus in contact with the glenoid, causing excessive stress. 24 Terzis et al considered lack of adequate muscle load on the scapula responsible for scapular growth limitations and showed that surgical reinnervation, especially reinnervation of the axillary nerve and the suprascapular nerve in Erb's paralysis, had positive effects on functional outcome as well as scapular growth. Functional improvements, measured as passive shoulder external rotation and abduction, were correlated to growth of scapular width and height. 38

In patients with total palsies, length discrepancies of the humerus, ulna, metacarpals, and phalanges between affected and unaffected side manifesting as limb length differences are common. The most frequent in patients with Erb's palsy, is discrepancy in the length of the humerus. 39 Some authors speculate that direct trauma to the shoulder joint at birth could be the reason for GHD. 15 40 Two MRI studies by van der Sluijs et al on young OBPP patients did not support the theory that deformity is caused by fracture to the proximal humeral epiphysies. 13 23 As far as we know, no study can accurately confirm or refute the theory of joint birth trauma as cause of GHD.

## Animal Models of GHD

Animal models of OBPP are useful for studying the natural course after OBPP since such studies are difficult to implement in patients. Recently, several studies on GHD and contractures have been conducted on murine models. Models involving surgical denervation and botulinum toxin-induced paralysis have been used. They reproduce the typical contractures seen in OBPP patients, but regarding GHD results are conflicting. 41 42 43 44 While the exact effects of botulinum toxin on development of muscle atrophy and other structural changes to the injected muscles are poorly understood, several factors, including duration of treatment, seem to interfere with sprouting, which is how functional recovery of neuromuscular transmission occurs following chemodenervation by botulinum toxin. 45

Some studies found these animal models suitable despite inconsistencies regarding glenoid version, humeral head version, and the area of humeral head flattening. 41 42 43 44 Soldado et al agree that the contractures induced resemble the clinical situation. However, they found a lack of posterior subluxation and glenoid retroversion in their study and considered the previous studies to do so as well, and therefore considered the established murine models unsatisfactory for GHD studies. 46

Li et al's first study measured glenoid version histologically and noted a glenoid anteversion. 41 However, in a follow-up MRI study, the result was retroversion. This is also the only study to have documented a significant glenoid retroversion in OBPP injured rats. 44 A difference in weight bearing patterns in quadruped animals, angle between the humerus and scapula, and possibly other unknown factors, could have affected outcome. 41 42 43 Kim et al found in a botulinum toxin-induced paralysis model in newborn mice that osseous deformities worsen with age and are irreversible after reapplication of muscle loading. 42 Botulinum toxin was injected to the supraspinatus muscle, primarily affecting this muscle even though the infraspinatus and posterior deltoid also were affected. In this study, botulinum toxin-induced imbalance lead to smaller humeral head volume, delayed mineralization, and smaller glenoid articular surface area and scapular area, showing that muscle imbalance could give rise to secondary bony deformities. There were trends toward normalization regarding the humeral head architecture with the shortest time of paralysis (2 weeks) and longest recovery time (14 weeks). Nevertheless, these parameters failed to recover to the level of the contralateral control side. Humeral version did not recover after discontinuation of botulinum toxin injections. This indicates that early recovery of normal muscle function and prevention of deformity establishment would be desirable. 42

Potter et al substantiates this finding in a similar study. Two weeks of botulinum toxin-induced paralysis of the supraspinatus muscle in mice was sufficient to cause irreversible reduction of humeral head size. Osteoclast numbers increased during recovery time after 2 weeks of paralysis, indicating increased bone remodeling. Continuous paralysis also increased osteoclast numbers. Contractures, however, were not assessed. 47 In an MRI study investigating ossification of the proximal humerus in patients with residual OBPP, Clarke et al discovered that the appearance of the greater tuberosity's ossific nucleus was delayed on the involved side. There was a trend toward delayed appearance of the ossific nucleus of the lesser tuberosity, and coalescence of the three ossific nuclei of the proximal humeral head as well. However, these two trends did not reach significance. In involved sides, the proportion of shoulders with ossification of the greater tuberosity varied significantly with glenoid type (concentric or nonconcentric as described by Pearl and Edgerton). 48

In a study on newborn rats, Crouch et al tested strength imbalance and impaired growth of denervated muscles on their own and in concert. 49 “Imbalance” was induced through botulinum toxin injections to the posterior aspect of the shoulder to paralyze the external rotators while “impaired growth” (i.e., denervation without imbalance) was obtained through neurectomy of the upper trunk as well as botulinum toxin injections to the anterior aspect of the shoulder. There was a “combined” group, which only did neurectomy, resulting in both muscle imbalance and impaired growth. The results showed that the difference in muscle mass between external and internal rotators was largest in the “imbalance” group. When measuring muscle mass of all muscles acting on the shoulder joint, the “impaired growth” and “combined” groups had the most loss of mass. Optimal muscle lengths for posterior deltoid, spindle deltoid (an external rotator found in rats), subscapularis, teres major, and long head of biceps were all correlated to osseous deformity. The authors concluded that impaired growth yields more pronounced glenohumeral joint deformity and contractures than imbalance. It should, however, be stated that, in the “impaired growth” group, the glenoid was anteverted, contrasting what is clinically noted. The biggest changes were decrease in glenoid inclination angle and inferior translation of the humeral head. These are not typically assessed clinical parameters. The authors also speculated that muscle imbalance could position muscles in a lengthened or shortened state, possibly affecting optimal muscle length and that passive stretching therefore could be beneficial for contracture prevention. 49

There have been computational studies to determine which muscles contribute to GHD and internal rotation contracture. Their findings show that muscle imbalance and/or impaired growth through the infraspinatus, subscapularis, latissimus dorsi, long head of biceps, anterior deltoid, pectoralis major, and long head of triceps are theoretically able to induce GHD. 50 Muscles were considered able to develop GHD if imbalance (i.e., the tested muscle was allowed to produce a force of 30% of its maximal active force while the other, unaffected muscles, were inactivated and only produced passive force) or impaired growth (i.e., the tested muscle's length was reduced with 30% while unaffected muscles had normal functional length), increased the posteriorly directed, compressive force on the glenoid. It was recommended that these muscles should be given extra attention in future studies on this subject. The subscapularis was noted to be the only muscle able to induce both GHD and shoulder contracture through both mechanisms. 50

In a follow-up study, a C5-C6 injury was simulated and muscles innervated by those nerve roots could, through muscle imbalance (i.e., affected muscles were given no resting tone while unaffected muscles were given 30% of maximal tone), impaired growth (i.e., affected muscles were simulated to be 30% shorter while unaffected muscles remained normal length) or a combined mechanism, affect the direction of shoulder joint forces and ROM. The contribution of C5-C6 to the subscapularis and teres major was considered unclear, and therefore two scenarios were tested. In the first, they were both considered affected. In the second, teres major and the lower belly of the subscapularis were regarded as unaffected. Supraspinatus and infraspinatus were always treated as affected. When the entire subscapularis was considered affected, the ROM was more restricted. All three scenarios yielded restricted ROM and posteriorly directed forces, which were sufficient to produce GHD. The combined group had the most profound effect, and impaired growth was found to contribute to a larger extent than muscle imbalance. 51

The key clinical studies cited are summarized in Table 2 .

**Table 2:** Summary of the key clinical studies cited

Author and title	Study design	Number of patients in the study, mean age	Included patients and study design
Hoeksma et al 2003. 11 Shoulder contracture and osseous deformity in obstetrical brachial plexus injuries	Retrospective	*n* = 67 Age = 3.7 (range: 1–7)	Contracture defined as decrease in passive range of motion > 10 degrees compared with healthy contralateral side. Osseous deformity defined as nonspherical humeral head or abnormal glenoid on radiograph
Iorio et al 2015. 12 Glenohumeral dysplasia following neonatal brachial plexus palsy: presentation and predictive features during infancy	Retrospective	*n* = 19 Age = 16 wk (14–46 interquartile range)	Children who underwent surgical exploration with preoperative MRI. GHD classified according to Waters et al
van der Sluijs et al 2004. 13 Secondary deformities of the shoulder in infants with an obstetrical brachial plexus lesions considered for neurosurgical treatment	Prospective	*n* = 26 Age = 5.6 mo (2.7–14.5)	Children with inadequate recovery of function in the first 3 mo of life and examined with MRI. Deformity defined as posterior subluxation, shape of glenoid as according to Birch, and glenoid retroversion
Kozin 2004. 19 Correlation between external rotation of the glenohumeral joint and deformity after brachial plexus birth palsy	N/A	*n* = 33 Age = 4.9 y (1.8–10.1)	Children with residual impairment in shoulder movements examined with MRI. Deformity defined as glenoid retroversion, posterior subluxation, and size of the humeral head
van der Sluijs et al 2001. 23 Deformities of the shoulder in infants younger than 12 mo with an obstetric lesion of the brachial plexus	Prospective	*n* = 16 Age = 5.2 mo (2.7–8.7)	Infants with inadequate recovery in the first 3 mo of life and therefore assessed with MRI. Assessed parameters were glenoid version, PHHA, and Birch classification
Pearl and Edgerton 1998. 21 Glenoid deformity secondary to brachial plexus birth palsy	N/A	*n * = 25 Age = range 1.5–13.5	Children undergoing secondary surgery due to internal rotation contracture. Deformity defined as abnormal glenoid shape on intraoperative arthrograms
Waters et al 2009. 27 Correlation of radiographic muscle cross-sectional area with glenohumeral deformity in children with brachial plexus birth palsy	Retrospective	*n* = 74 Age = 2.5 y (7 mo–6.2 y)	Children with different degrees of GHD according to Water's classification and previous MRI scans. Cross-sectional area of shoulder muscles assessed on MRI
van Gelein Vitringa et al 2009. 28 An MRI study on the relations between muscle atrophy, shoulder function, and glenohumeral deformity in shoulders of children with obstetric brachial plexus injury	Prospective	*n* = 24 Age = 3.3 y (14.7 mo–7.3 y)	Children with internal rotation contracture undergoing MRI scan. Glenoid version, Birch classification, and posterior subluxation were the assessed deformity parameters. Atrophy assessed on MRI by measuring muscle thickness and calculating volume
Pöyhiä et al 2005. 18 MRI of rotator cuff muscle atrophy in relation to glenohumeral joint incongruence in brachial plexus *birth injury*	Prospective	*n* = 39 Age = 7.7 y (range 2.0–15.7)	Children undergoing MRI due to internal rotation contracture, absent active external rotation, or possible posterior subluxation. Both nerve reconstructed ( *n* = 7) and secondary subscapularis released patients ( *n* = 6) were included. Glenoid version, glenoid configuration, and PHHA were assessed. Atrophy assessed as decrease in muscular thickness on MRI
Hogendoorn et al 2010. 26 Structural changes in muscle and glenohumeral joint deformity in neonatal brachial plexus palsy	Prospective	*n* = 102 Age 5.0 (±3.4 y)	Patients with passive external rotation < 0 degree or poor Mallet score who underwent MRI. Both primary nerve reconstructed and conservatively treated patients. Glenoid shape, glenoid version, and PHHA assessed on MRI. Muscle atrophy defined as decreased thickness diameter compared with contralateral side
van Gelein Vitringa et al 2013. 34 Scapular deformity in obstetric brachial plexus palsy and the Hueter–Volkmann law; a retrospective study	Retrospective	*n* = 58 Age = 20 mo (1–88 mo)	Children with unilateral OBPP, Narakas grade I–III, and MRI of the shoulders. PHHA compared with scapular measurements
Pearl et al 2013. 24 Geometry of the proximal humeral articular surface in young children: a study to define normal and analyze the dysplasia due to brachial plexus birth palsy	Retrospective	*n* = 25 Age = 3.7 y (±3.1)	Children with internal rotation contracture of 0 degree or less underwent MRI. Degree of contracture correlated to humeral head symmetry and glenoid dysplasia
Terzis et al 2014. 38 Morphometric analysis of the association of primary shoulder reconstruction procedures with scapular growth in obstetric brachial plexus paralysis patients	Retrospective	*n * = 73 Age = N/A	Children with Narakas grade I–IV with different treatment strategies. Investigating association between OBPP severity, type of intervention, and scapular dimensions assessed on X-rays
Al-Qattan 2003. 56 Obstetric brachial plexus palsy associated with breech delivery	Retrospective	*n* = 28 Age = N/A	All patients with OBPP delivered with breech delivery at one medical center. Surgical exploration of patients unable to actively flex the elbow joint at 4 mo of age
van Gelein Vitringa et al 2015. 58 Degree of contracture related to residual muscle shoulder strength in children with obstetric brachial plexus lesions	Prospective	*n* = 34 Age = 10 y	Patients with Narakas grade I–III. External and internal shoulder rotation' strength measured with hand held dynamometer. Contracture measured passively in external and internal rotation of the shoulder
Einarsson et al 2008. 30 Subscapularis muscle mechanics in children with obstetric brachial plexus palsy	Prospective	*n* = 9 Age = 6 y (range 1–10)	Mechanical testing and assessment of histological features of subscapularis muscle biopsies in children with OBPP associated internal rotation contracture undergoing subscapularis lengthening. Controls ( *n* = 7) were healthy individuals aged 24 y (15–33) with muscle samples from different upper limb muscles
Hultgren et al 2010. 31 Structural characteristics of the subscapularis muscle in children with medial rotation contracture of the shoulder after obstetric brachial plexus injury	Prospective	*n* = 13 Age = 74 mo (11–186 mo)	Children undergoing corrective surgery due to OBPP associated internal rotation contracture with intraoperative subscapularis muscle biopsies. Histological assessment of these biopsies. No control biopsies

Abbreviations: GHD, glenohumeral joint dysplasia; MRI, magnetic resonance imaging; OBPP, obstetric brachial plexus palsy; PHHA, percentage of humeral head anterior to the middle of the glenoid fossa.

## Theories Regarding Pathogenesis of Contractures

In rats, Soldado et al showed that cutting the suprascapular nerve (denervation of infraspinatus and supraspinatus muscles) without taking the subscapular nerve (intact subscapularis muscle innervation) lead to atrophy of the subscapularis muscle and to internal rotation contacture. 33

Another study supporting the role of muscle imbalance as cause of contractures found that botulinum toxin-induced paralysis of the external rotators led to internal rotation contracture. In this study, contractures could be resolved, but only after early cessation of botulinum toxin injections. Two weeks of botulinum toxin injections followed by 14 weeks of recovery time without injections could reverse shoulder contracture. However, if injections were allowed to go on for 4 weeks with 12 weeks of recovery time, there was impairment of shoulder external rotation at the endpoint at 16 weeks. 42

Contrasting these findings, Nikolaou et al showed that, in mice, excision of external rotator muscles and elbow flexors did not result in contracture formation or muscular shortening. 52 This finding is supported by a later study proving that excision of elbow extensors did not lead to elbow flexion contracture. 53 Nikolaou's C5, C6 neurectomy group did, however, develop shoulder internal rotation and elbow flexion contractures. The neurectomy group also expressed impaired longitudinal growth of the biceps, brachialis, and subscapularis. The subscapularis shortening, measured as sarcomere lengths, correlated to shoulder internal rotation contracture. Impaired growth of elbow flexors was not likely to be caused by lack of passive stretch since elbow extension was preserved. The elbow contractures were completely resolved after resection of biceps and brachialis muscles. In this study, fibrosis or loss of myogenic stem cells was not the cause of muscular shortening or contractures. 52 In a follow-up study, Nikolaou et al corroborated the finding that muscle fibrosis alone cannot explain the impaired growth. The less fibrotic brachialis muscle contributed more to elbow contracture than the more fibrotic biceps, and contracture preceded fibrosis. 54

In another mouse study, Weekley et al demonstrated that elbow flexion contracture was not influenced by muscle imbalance. Triceps tenotomy, in concert with denervation, did not lead to worse contracture or biceps/brachialis shortening than denervation alone. Triceps tenotomy alone did not give rise to elbow flexion contracture or muscle shortening of biceps and brachialis. Instead, denervation and time to reinnervation were the key factors deciding severity of contracture. Less regenerating axons in the musculocutaneous nerve lead to worse contractures. Biceps and brachialis sarcomere length correlated to the degree of contracture. Longer sarcomeres indicating shorter functional muscle length and the shortening of the biceps and brachialis were correlated to the degree of elbow flexion contracture. The degree of elbow contracture did not differ between C5-C6 excision and total excision from C5 to T1. 53

In clinical studies, results are conflicting. In one, a higher Narakas classification was linked to worse elbow flexion contracture. 55 In another, no such correlation could be established. 14 Al-Qattan noted a striking absence of shoulder and elbow contractures in the rare group of OBPP associated with breech delivery. 56 Eight of the breech delivery patients presented late to the clinic (2–16 years) and despite limitations of active shoulder movement, no decrease in passive shoulder external rotation or elbow extension was seen. Five limbs met the inclusion criteria for surgical exploration. Of 11 intraoperatively assessed nerve roots, 10 were avulsed preganglionically with only one postganglionic avulsion.

To our knowledge, the loss of muscle strength in the usual postganglionic injury is comparable to the unusual preganglionic. Therefore, lack of muscle imbalance as the reason for preserved ROM seems unlikely. A mouse model of preganglionic injury agrees with this clinical observation. Despite having equal atrophy and denervation of the musculocutaneous nerve, as measured immunohistochemically, the preganglionic group had less severe contractures. The proposed mechanism for this is preservation of afferent innervation keeping muscles connected to the dorsal root ganglion. Muscle spindles, a stretch sensing organ in the muscles, are intact and preserve the signaling of the growth hormone ErbB. Signaling was increased in both the injury groups suggesting, but not proving, ErbB could have a causative role in contracture formation. 57

To test if muscle imbalance impacts passive ROM, van Gelein Vitringa et al tested external and internal shoulder rotation strength and the passive range of rotational motion in a group of 34 OBPP patients, Narakas I to III, and mean age 10 years old. As expected, the affected shoulder was weaker than the contralateral control in both directions but, surprisingly, equally reduced in external and internal rotation. Muscle imbalance could not be found. No correlation between residual muscle strength and shoulder contracture, or the ratio between external and internal rotation strength and shoulder contracture, were established. 58

Other studies reached conflicting conclusions. One did find an imbalance and a correlation between shoulder external rotation strength and active external ROM. 59 Another study found the shoulder external rotators to be weaker than the internal rotators. However, elbow flexion contracture was the most frequent ROM deficit in this study, even though elbow flexion strength was more impaired than extension strength as compared with the unaffected contralateral arm. 60 This implies that other factors than muscle imbalance caused the contracture.

## Conclusion

While there is a vast clinical knowledge about treatment of complications after OBPP, 10 61 62 the research focus shifted in the recent years to the etiology of GHD and shoulder contractures.

Due to the close links between contracture formation and GHD, it is reasonable to assume that both, to a substantial extent, share causative factors and it is probable that GHD is caused by abnormal posture related to contractures. However, it has been shown that one can exist without the other, making individual factors plausible as well. Based on the existing knowledge, we do not believe muscle imbalance to be the only factor generating the contractures and osseous deformities. The denervation induced cellular alterations that lead to impaired growth seem to be a major influencer. Currently, these muscle alterations on a cellular level remain largely undiscovered. The muscle imbalance, and the abnormal mechanical loading on bony structures which it causes, could presumably be alleviated by early reinnervation of target muscles. While this probably would diminish denervation-induced growth impairment as well, we hope future studies can identify injury-inducing pathways for alternative treatment strategies.
